# Lens Material of the Eyelid Masquerading as Phakomatous Choristoma After Cataract Surgery

**DOI:** 10.7759/cureus.38718

**Published:** 2023-05-08

**Authors:** Keiko Inouye, Harris Ahmed, Soungmin Cho, Douglas Van Putten

**Affiliations:** 1 Ophthalmology, College of Osteopathic Medicine, Western University, Pomona, USA; 2 Ophthalmology, Loma Linda University Medical Center, Loma Linda, USA

**Keywords:** ophthalmic pathology, retained lens material, lens capsule, cataract surgery, phakomatous choristoma

## Abstract

Cataract surgery is the most commonly performed surgery worldwide. While retained lens fragments after cataract surgery are common, to our knowledge, there is no prior case report of the lens material being deposited outside of the eye. Here, we present a case of an elderly patient with an upper eyelid lesion containing a fragment of the basement membrane and proteinaceous lens-like material, initially mistaken as phakomatous choristoma. Phakomatous choristoma is a type of benign congenital tumor consisting of lens tissue, which is thought to be secondary to aberrant migration during lens formation. Upon further review, it was later confirmed to be postoperative capsular material embedded into the eyelid.

## Introduction

Cataract surgery is the most commonly performed procedure worldwide with an estimated 20 million procedures done annually [1]. Complications of cataract surgery include posterior capsular rupture with vitreous loss, dropped or retained crystalline lens, infection, and inflammation. Retained lens fragments after cataract surgery can lead to a severe inflammatory reaction, increased intraocular pressure, macular or corneal edema, or even permanent vision loss [2,3]. Retained lens fragments are usually identified within days of the postoperative period, although there are reports of patients presenting with retained lens fragments as late as 65 years after the procedure [4].

In this report, we discuss a patient presenting approximately two years after surgery with a suspected chalazion that, upon biopsy, contained proteinaceous material resembling a crystalline lens and a fragment of smooth basement membrane resembling a lens capsule.

## Case presentation

**Figure 1:**
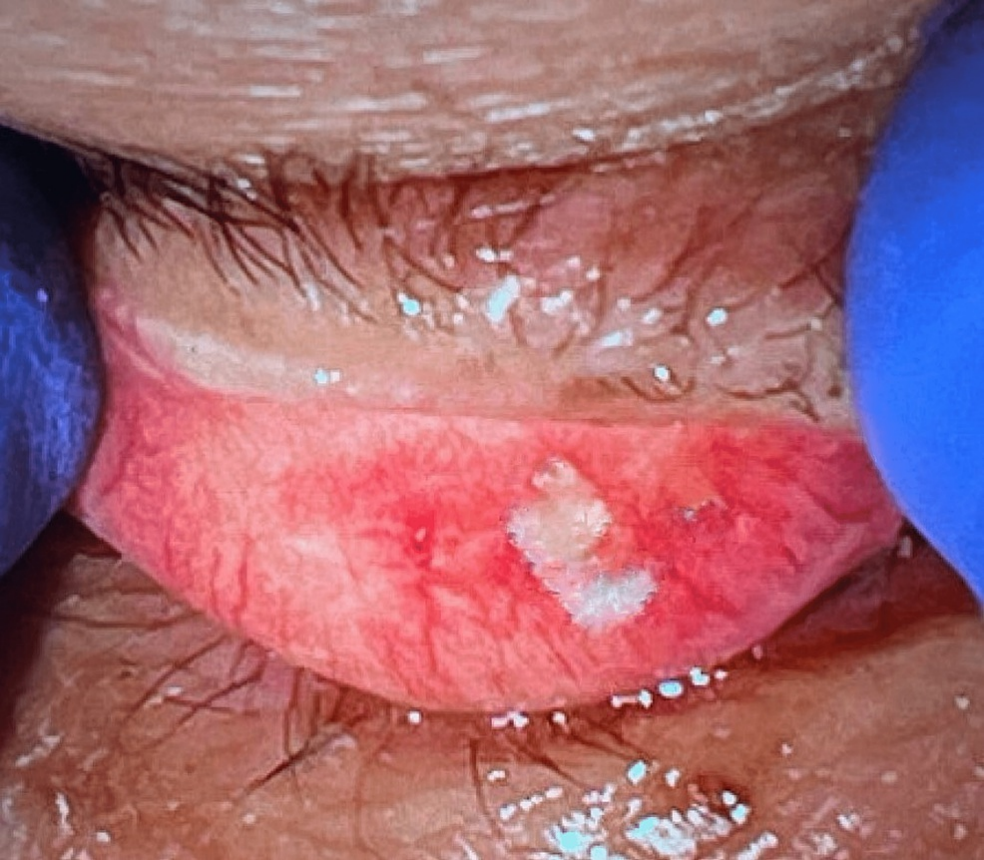
External photograph of left upper eyelid eversion demonstrating a flat, irregular yellow lesion.

An 88-year-old female presented to the office after referral for a suspected chalazion on the temporal palpebral conjunctiva of the left upper eyelid. The cataract surgery was performed by phacoemulsification, and there were no significant intraoperative or postoperative complications. The patient presented to the clinic two years later for a gradually worsening foreign body sensation and tearing. Everted eyelid examination showed a whitish material on the lateral aspect of the upper eyelid tarsal conjunctiva, concerning for a foreign body or chalazion. The patient subsequently underwent complete excision in-office using an 11 blade, and the specimen was sent to pathology to rule out possible malignancy (Figure 1). The patient tolerated the procedure well and there were no postoperative complications at the one-month follow-up visit.

**Figure 2:**
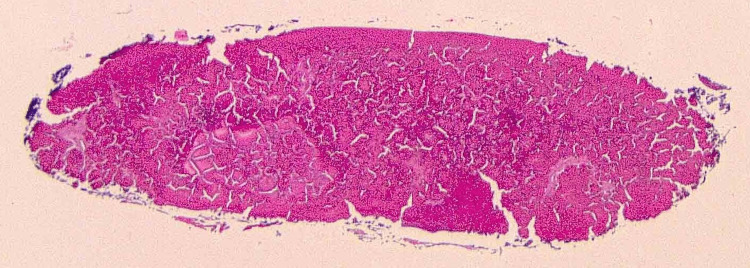
Microscopic examination of the collected specimen showing disc-shaped densely packed protein material resembling a small native lens.

**Figure 3:**
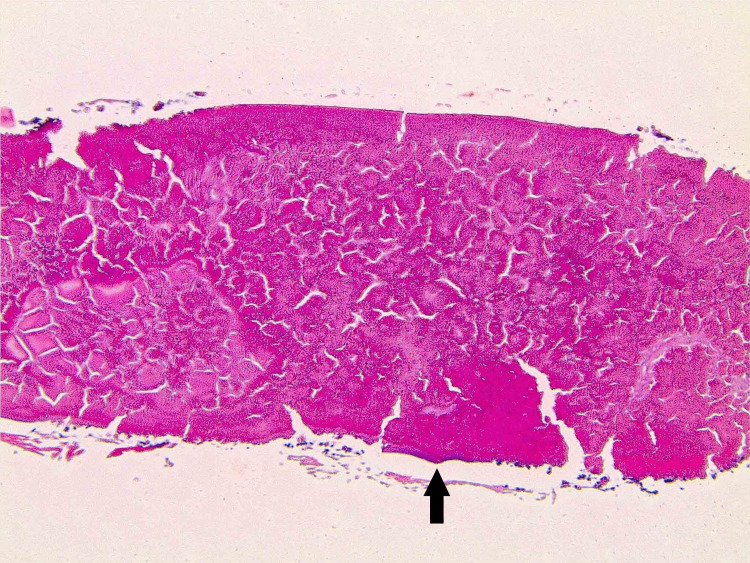
High-power view of the collected specimen showing a fragment of smooth basement membrane (as indicated by the arrow) reminiscent of lens capsule on microscopic examination.

On gross examination, the specimen weighed less than 1 gram, was approximately 2 × 2 × 1 mm, and was red-tan in color. Histological examination revealed a disc-shaped densely packed protein material resembling a small native lens (Figure 2). High-power view showed a fragment of smooth basement membrane partially encompassing the proteinaceous material, reminiscent of a lens capsule (Figure 3).

MRI of the brain was ordered as part of the initial evaluation to rule out other or concurrent orbital or extraocular lesions. This imaging revealed moderate cerebral atrophy and non-specific white matter changes as well as changes possibly related to chronic hypertension, but no periocular or orbital lesions were present.

## Discussion

The patient was originally referred for a suspected chalazion. Chalazia generally presents on the upper eyelid due to the increased concentration of meibomian glands on the tarsal plate of the upper lid. Like this patient, those presenting with chalazia may complain of nonspecific symptoms such as discomfort or a foreign body sensation [5].

The specimen collected during biopsy revealed densely packed proteinaceous material resembling a crystalline lens encompassed by a small fragment of the basement membrane, reminiscent of phakomatous choristoma. Although phakomatous choristoma stains similar to lens tissue and epithelium, they do not take on the shape of a crystalline lens and are not composed of densely packed proteinaceous material. These tumors present on H&E staining with aggregates of cuboidal cells with surrounding pseudo-glandular structures and a characteristic periodic-acid-Schiff (PAS)-positive basement membrane surrounding the ectopic lens cells, resembling a lens capsule. Scarcities of cytoplasmic organelles and “bladder” or Wedl cells, which are found in cataractous lenses, have been noted [6-9]. Immunohistochemistry will stain positive for S-100 as well as crystalline and vimentin, which are specific to human lenticular tissue. They also stain negative for cytokeratin, which is present in other epithelial cells throughout the body [10,11]. Neuron-specific enolase, which has moderate activity in lens epithelium during the early stages of development, is found to be positive in these tumors [12,13]. Additionally, phakomatous choristoma usually involves the inferomedial eyelid or the anterior orbit and not the upper eyelid [7].

In this case, further review by an ophthalmic pathologist revealed that it was truly an unusual presentation of phakomatous choristoma given the patient’s older age. The external photograph of the everted upper lid was also provided to the pathologist**. **Given its superficial location, patient age, and history, it was decided that this was more consistent with retained anterior capsule embedded into the tarsal conjunctiva following prior cataract surgery, rather than phakomatous choristoma. Other differential diagnoses were considered prior to the pathology review, such as chalazion, retained foreign body, and malignancy such as sebaceous cell carcinoma. However, these diagnoses were less likely given the lack of significant inflammation that would suggest either chalazion or retained foreign body. Additionally, there were no obvious foreign bodies noted on the pathology review. Sebaceous cell carcinoma was also less likely given that no obvious undifferentiated sebaceous cells with high mitotic activities were seen on the pathology specimen.Since the procedure was reportedly uncomplicated, it was theorized that the tissue removed from the anterior capsule following the capsulorhexis was deposited along the conjunctiva and eventually embedded as it healed. Additionally, the use of a wire versus plate speculum could have allowed the tissue to deposit in the lid due to the exposed conjunctiva. There is one other reported case of retained capsular material following uncomplicated cataract surgery. In this case, the capsule adhered to the anterior chamber of the eye, masquerading as Descemet’s membrane detachment, and presented with corneal edema and decreased visual acuity [14].

Retained fragments of lens tissue are rare complications of cataract surgery. They occur most commonly due to low levels of surgical experience or complicated cases [15]. Smaller retained fragments or nuclear tissue may be treated conservatively with observation and corticosteroids. However, larger fragments are surgically removed. Depending on the location of the fragment, greater measures may be taken for removal; fragments depositing in the back of the eye often require vitrectomy to remove them [3]. In order to prevent retained fragments, measures must be taken to ensure that the nuclear material is cleared completely. Patients with smaller pupils are at higher risk of fragments depositing between the anterior capsule and the posterior iris [16]. Retained fragments within the eye commonly present with irritation and edema. Even with the nuclear tissue being deposited outside of the eye, our patient presented with irritation and foreign body sensation. If left untreated, retained nuclear material can result in permanent vision loss.

## Conclusions

Until now, there have been no reported cases of lens or capsular tissue depositing outside of the eye following cataract surgery. The tissue was likely deposited following the capsulorhexis in an otherwise uncomplicated procedure. For patients presenting with lesions of the upper eyelid following cataract surgery, embedded capsular tissue should be included on a list of possible diagnoses coupled with thorough history taking and slit lamp examinations.
